# Gaze Parameters in the Analysis of Ambiguous Geometric Shapes

**DOI:** 10.1177/2041669521998392

**Published:** 2021-03-12

**Authors:** Linda Krauze, Ilze Ceple, Jurgis Skilters, Mara Delesa–Velina, Baingio Pinna, Gunta Krumina

**Affiliations:** Department of Optometry and Vision Science, Faculty of Physics, Mathematics and Optometry, University of Latvia, Riga, Latvia; Laboratory for Perceptual and Cognitive Systems, Faculty of Computing, University of Latvia, Riga, Latvia; Department of Mathematics, Faculty of Physics, Mathematics and Optometry, University of Latvia, Riga, Latvia; Department of Biomedical Sciences, University of Sassari, Sassari, Italy; Department of Optometry and Vision Science, Faculty of Physics, Mathematics and Optometry, University of Latvia, Riga, Latvia

**Keywords:** visual grouping, gaze, shape perception, eye movement analysis

## Abstract

This study explores perceptual organisation and shape perception when viewing a tetragon and an additional element (a dot) that is located at varying positions and distances next to the tetragon. The aim of the study is to determine the factors that can alter the interpretation of object configuration and impact whether the presented tetragon is perceived as a diamond or a square. Methods used in this study are a forced-choice task as a subjective measurement and eye tracking as an objective measurement of perceptual processes. Overall, 31 stimuli were presented to the participants: a tetragon in two different sizes with an additional element (a dot) located inside or outside the object at three different positions at three distances. The results indicate significant changes in shape perception, depending on the location of the additional element. The results are complemented with eye movement analysis indicating that as the distance between the elements increases, there is a higher probability of either of the two shape interpretations and the gaze is less likely to be directed to the area between the stimuli. Furthermore, the subjective perception of shape is codetermined by the shape perception when the tetragon is presented without the additional element.

## Introduction

Perceptual organisation is the key for constructing a coherent visual percept of the external environment. It refers not only to the way we process sensory input based on the levels of luminance or separate shapes, edges, and lines but also to the way we segment this information, by creating distinct objects or groups of objects in our percept.

The questions regarding why we see the world exactly as we see it, what determines the processing of visual information and how objects and their forms are perceived and analysed have been extensively studied before (see Palmer, 1999; Wagemans, 2015 for overviews). The principles of visual grouping were first described by Max Wertheimer (1923) at the beginning of the last century. Since then different studies have demonstrated principles underlying the ways individual elements are grouped into larger wholes (Pinna, 2010, 2015; Wagemans et al., 2012). Theories of visual grouping describe how humans are naturally inclined to understand objects as an entire structure rather than the sum of objects’ parts (Pohl, 2016). The objects may be perceived only after the process of figure-ground segregation as the visual field requires a relatively strong differentiation between the objects and their background so that objects and their details can be grouped together based on the principles of visual grouping (Wertheimer, 1923). Gestalt principles of visual grouping provide important guidance on what element properties enable perception of wholes: these properties include proximity, similarity, good continuation, closure, convexity, symmetry, as well as past experience (Brooks, 2015).

Principles of grouping are also important when defining the shape of an object and assigning the meaning to it. Figure-ground organisation (or segmentation) is the core process of object form assignment and is based on the surroundedness, size, orientation, contrast, symmetry, convexity, and parallelism of the object (Pinna, 2010). Theories of figure-ground organisation state that elements are grouped together within the framework of perceptual and visual information grouping and that distinct boundaries are perceived as a figure with a certain shape, while the surrounding elements are perceived as the background (Peterson & Salvagio, 2010). However, visual perception is more than visual grouping and segmentation; it also extends to the assigning of meanings to the different shapes, thus creating the complex world that is perceived in everyday life (Pinna, 2010).

Pinna (2015) demonstrated that visual grouping can also affect shape perception which can easily be altered by changing the location of a black dot located close to an object. By adding an additional element to a tetragon, either next to the corner or one side of the tetragon, perception of the shape can be altered between a diamond and a square. This study aims to expand the results of Pinna (2015) by using (a) eye-tracking methodology and (b) a forced-choice task in analysis of additional variables (stimulus size, distance, and location of the additional element in respect to the main shape) affecting the processes of shape perception. We assume that the processes of visual grouping at least to some extent impact the resulting shape perception.

### Visual Grouping and Eye Movements

Our visual system captures images of the environment during short fixations located at different regions of the visual field. A common assumption is that we perceive visual information through a series of saccadic eye movements that are interrupted by visual fixations during which visual information is perceived. Although most of our fixations are located on certain objects that draw our attention, different studies (Findlay, 1982; Van der Stigchel & Nijboer, 2011; Venini et al., 2014) have demonstrated that we frequently make fixations at locations that are between objects, that is, in empty space. Eye movements of this type have been described as centre of gravity (COG) fixations, averaging saccades, or global effect, and they are located in the geometric centre of the available visual elements. The studies on global effect of saccadic eye movements have led to the point of view that COG fixations reflect simultaneous encoding and perceiving of multiple stimuli, allowing more efficient and faster processing of task-related items (Venini et al., 2014). COG fixations occur only when the target and distractor are located relatively close to one another so that the objects fit into the visual field. Distance between the objects must subtend a visual angle of less than 30° because only then objects can be perceived together without additional eye movements (Walker et al., 1997). Fixations in the neighbourhood of the COG rather than precise fixations to target or distractor position were initially attributed to the poor spatial resolution of an early saccade targeting mechanism. However, it may confer advantages in visual search tasks and could be used mostly for strategical purposes, especially in tasks with many distractors.

Fixations in the COG are more common for observers who have previous experience performing similar tasks than for beginners. These types of fixation reduce the total number of necessary fixations and decrease saccadic eye movement latency (Venini et al., 2014). Although it has been suggested that saccades are directed towards the COG, the analogy that gaze is centred between the visual elements cannot be applied in all cases. The need to direct the saccade towards the COG depends on the visual characteristics of the objects, such as the size of the stimuli and distance between them, as well as the instructions given to the observer. If stimuli have different sizes, then the COG and fixation is located towards the largest object (Findlay, 1982; He & Kowler, 1991).

Gaze parameters such as direction, fixation duration, and so on, serve as measurements of observers’ overt attention and can be attributed important roles in determining different top-down and bottom-up factors that guide our eye movements and thus form our visual perception (Kulke et al., 2016; Sheliga et al., 1994).

The impact of visual grouping on shape perception has been extensively studied before (e.g., Peterson & Salvagio, 2010; Wagemans et al., 2012), but in addition to testing different parameters affecting shape perception, we also aim to link the direct stimulus-driven attentional processes in perceptual grouping to gaze parameters. We hypothesise (a) that the location of the additional element and the size of the stimulus determine the perception of the shape as diamond or square, (b) that in the process of visual grouping eye movement analysis indicates that the gaze is directed towards the space between the objects, and (c) that the initial interpretation (without the additional element) determines the further shape assignment (diamond or square) when presented with the additional element.

## Method

The study was divided into two parts: a forced-choice task (Experiment 1) in which the subjective percept of object shape was determined and an eye-tracking task (Experiment 2) which was used as an objective measurement of the attentional processes of the observer.

### Participants

Overall, 103 participants took part in the study. All participants had normal or corrected-to-normal vision. Participants were divided into two groups. The first group (*Experiment 1*) consisted of 86 participants (72 females and 14 males, 23 ± 5 years old) who performed a forced-choice task and the second group (*Experiment 2*) consisted of 17 participants (10 females and 7 males, 25 ± 6 years old) who performed the eye-tracking task.

All participants provided written informed consent for participation in the study. Participants were informed of the overall purpose of the study and their rights to stop the experiment at any time.

## Experiment 1

### Stimuli

**Figure 1.**
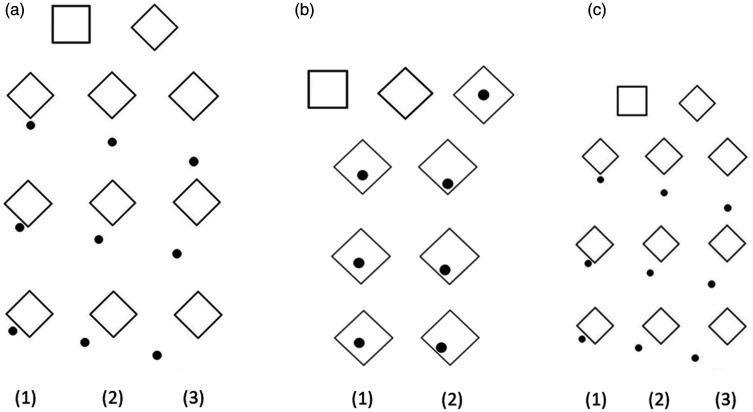
Stimuli demonstrated in Part A (A), Part B (B) and in Part C (C). For each part of the experiment, the control stimuli are presented in the first row, while the stimuli with the additional element located in vertex, middle, and edge positions are represented in the second, third, and fourth rows, respectively. The additional element was located in one of the three distances from the tetragon: closest (1), the middle (2), and the farthest (3; Parts A and C). In Part B, the additional element was located in one of the two distances: closer to centre (1) or farther from centre (2).

A total of 31 black on white stimuli (see Figure 1) were created in two different sizes and presented to the participants in three parts (A, B, and C) of Experiment 1. The stimuli in each of three parts of the experiment were demonstrated in randomised order. In each part of the experiment, participants were presented with a tetragon without any additional element (*square*), and with its rotated version (*diamond*). As these two figures did not incorporate the additional element and hence no distance and no position measure, we will refer to them as control stimuli in the further data analysis.

**Figure 2.**
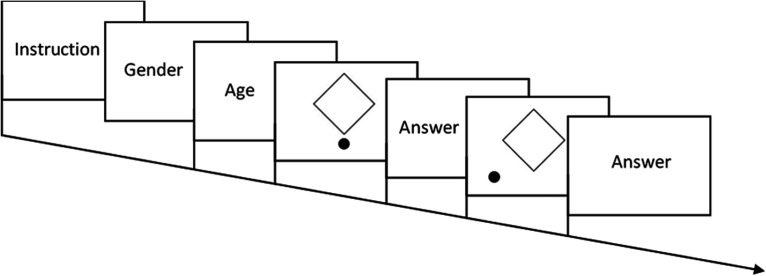
Schematic representation of Experiment 1 sequence.

In *Part A*, participants were presented with a tetragon with an additional element (a dot) located at the vertex or edge or the middle (between these two positions) at three different distances (0.4°, 2.1°, and 3.9°; i.e., the distance between the circumference of the tetragon and the circumference of the additional element) outside the figure, in addition to the control stimuli (Figure 1A). In *Part B*, a tetragon with an additional element (a dot) that was located inside the figure in three different positions and at two different distances (2.1°: Figure 1B-1 and 0.4°: Figure 1B-2; from the bottom vertex or from side of the tetragon) was presented to the participants (Figure 1B). Note that an additional control figure, a tetragon with a dot in its centre, was included in Part B. In Part A and Part B, the diagonal of each figure without the additional element was 5°, and the diameter of the additional element was 0.9°. In *Part C*, the same stimuli as in Part A only smaller in size were presented to the participants. The diagonal of the figure without the additional element was 2.6°, and diameter of the additional element was 0.5°, so that the whole stimulus size was not larger than 5°. The distance between the figure and the additional element was 0.2°, 1.1°, and 2.0°. Line thickness was 0.1° for all stimuli in all three parts of Experiment 1.

Stimulus parameters used in all parts of the study are summarised in Table 1. The stimuli were presented on a 47.1 × 29.5 cm Dell monitor (P2213T; 1680 × 1050 px) at 65 cm distance from participant to the screen.

**Table 1.** Parameters of Stimuli Used in All Parts of Experiment 1 and Experiment 2.

	Part A	Part B	Part C
Number of stimuli	11 (9—with additional element, 2—control stimuli)	9 (6—with additional element, 3—control stimuli)	11 (9—with additional element, 2—control stimuli)
Stimulus properties	Additional element **outside** the figure;	Additional element **inside** the figure;	Additional element **outside **the figure;
Angular size of the whole stimulus	**>5°**	**5°**	**<5°**
Diagonal of the tetragon	5°	5°	2.6°
Diameter of the additional element	0.9°	0.9°	0.5°
Additional element position	Edge	Edge	Edge
	Vertex	Vertex	Vertex
	Middle	Middle	Middle
Distance	Closest (1)	Closer to centre (1)	Closest (1)
	Middle (2)	Farther from centre (2)	Middle (2)
	Farthest (3)		Farthest (3)

*Note: *The text in bold indicates the most important differences in the stimulus parameters, whose effects on shape perception and eye movements were studied in this study.

### Method

Participants performed a forced-choice task in which they were asked to report the percept of the shape of the demonstrated figure (i.e., whether they perceived the presented tetragon as a square or a diamond). The stimuli were presented using the QuestionPro (*Survey Analytics LLC, Seattle, WA*) online survey software and all stimuli (including the control stimuli) were presented in a randomised order. The control stimuli were presented interspersed between the other stimuli in the presentation sequence so that the percept and decision of the control stimuli does not affect the on-going analysis of the other stimuli. There was no correct answer to any of the presented stimuli, as each stimulus could be perceived as either of the two shapes. Each stimulus was presented separately for an unlimited time until the participants gave their response. Prior to the stimulus presentation, participants were required to provide information on their gender and age (see Figure 2).

### Results

An overview of the results for the shape perception task is given in Table 2. Table 2 summarises the shape perception responses in Experiment 1, giving the proportion of the participants who perceived the demonstrated tetragons as diamonds, as opposed to as squares.

**Table 2.** Percentage Distribution of Shape Perception Responses Perceiving Stimuli as Diamonds in Experiment 1.

Additional element position	Distance	Part A	Part B	Part C
Control^a^		46.5%	49.2%	47.3%
Square		4.7%	1.6%	3.6%
Vertex	Closest	60.5%	60.7%	65.5%
	Middle	57.0%	—	56.4%
	Farthest	50.0%	57.4%	49.1%
Edge	Closest	39.5%	44.3%	36.4%
	Middle	48.8%	—	40.0%
	Farthest	45.3%	52.5%	43.6%
Middle	Closest	47.7%	50.8%	49.1%
	Middle	57.0%	—	58.2%
	Farthest	52.3%	57.4%	41.8%

^a^Rotated tetragon without an additional element for Parts A and C; with an additional element in the centre of the figure in Part B.

Although several control stimuli were demonstrated in each part of the experiment, regarding the control figures, most of the participants did not consider the square as a diamond (1.6%–4.7%). Thus, this stimulus was dropped from the further data analysis and only data on the shape perception of the rotated tetragon without an additional element (Parts A and C) and of the rotated tetragon with an additional element in the centre of the figure (Part B) were included as the control stimuli.

**Figure 3.**
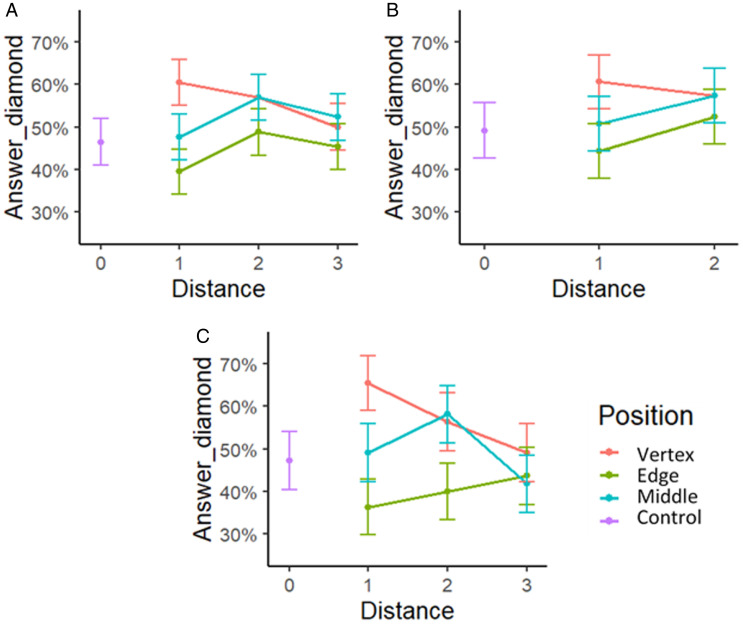
Shape perception responses obtained in Experiment 1 Part A (A), Part B (B), and Part C (C). The value of 100% would mean that all participants perceive the figure as a diamond. The value of 0% would mean that all participants perceive the figure as a square. The graph demonstrates the responses of the perception of shape depending on (a) the position of the additional element and (b) the distance—the nearest (1), middle (2), and farthest (3).

The results seem to demonstrate that (a) an additional element located either inside or outside the figure changes the perception of figure shape and (b) the perception of the diamond is more likely to occur if the additional element is located closer to the vertex of the figure. When the additional element is closer to the edge of the figure, the figure is more frequently perceived as a square. Furthermore, as the distance between the additional element and the line of the figure increases, the possibility of the figure being perceived as a diamond or as a square also changes. Figure 3 demonstrates how the responses of shape perception obtained in the forced-choice task change based on the distance of the element: the further the element is located, the less differences are observed in the shape perception of elements in different positions. In Part C, when the additional element is located at either the vertex or the edges of the stimulus (not the middle position), the changes of the response based on object distance are actually linear.

To test our findings, we analysed the original shape perception data using the mixed effects logistic regression model. Specifically, each participant’s response on each stimulus was included as an observation in the model, where the response *diamond* was coded with 1, and the response *square* was coded with 0.

The full model included distance, position type, and control shape perception as fixed effects, and survey participant as a random effect. The likelihood ratio test for the difference of the deviance scores between the nested models was used to determine the significance of the model terms. A separate model was fit for each of the experiment Parts A, B, and C.

For experiment Part A, there was a significant main effect for the control shape perception, χ^2^(1) = 79.1, *p* < .001, and for the position, χ^2^(2) = 17.3, *p* = .002, but there was no significant main effect for the distance, χ^2^(1) = 0, *p* = 1.000. There was one significant interaction between the distance and position, χ^2^(2) = 7.49, *p* = .024. Analysing the contrasts of the position effect, it was revealed that the difference in shape perception between the vertex and edge positions was significant (*p* < .001), while the difference between the vertex and middle position was not (*p* = .220). Further examination of the interaction effect showed that the distance effect on the shape perception was significant only when comparing the answers for the stimuli with additional elements at the vertex and edge positions (*p* = .015) but not between the vertex and middle positions (*p* = .22). Namely, the closer the additional element was located to the main stimulus, the greater on average were the shape perception differences between these two position types.

The results were very similar for Part C: There was a significant main effect for the control shape perception, χ^2^(1) = 42.72, *p* < .001, and for the position, χ^2^(2) = 18.02, *p* < .001, but no significant main effect for the distance, χ^2^(1) = 1.91, *p* = .167. There was a significant interaction between the distance and position, χ^2^(2) = 6.05, *p* = .048. A slight difference compared with experiment Part A was revealed when analysing the contrasts. Specifically, there was a significant difference in shape perception both between the vertex position and the edge position, *p* < .001, and between the vertex and the middle position, *p* =.021. When analysing the interaction effect, the result was the same as for experiment Part A: the distance effect was important only when comparing the results for the additional elements at the vertex and edge positions (*p* =.016).

For experiment Part B, there were again significant main effects for the control shape perception, χ^2^(1) = 44.25, *p* < .001, and the position, χ^2^(2) = 6.79, *p* = .034, and no significant main effect for the distance, *p* = .247. However, no interaction effects were revealed for experiment Part B. The contrast analysis showed that there was a significant difference between the edge and the vertex positions, *p* = .011, but no difference between the vertex and the middle position, *p* =.161.

To sum up the results, all three parts of the experiment (A, B, and C) revealed a significant difference in shape perception when the additional element was located either at the vertex or the edge of the figure. The distance effect was important only in interaction with the position and only when the additional element was located outside the tetragon (Parts A and C). The shape perception answers for most of the situations are not significantly different for the stimulus with the additional element in the edge and the middle positions (except for the Part C). This can be seen also in Figure 3, where the blue lines corresponding to the middle position figures are rather close to the green lines corresponding to the edge position figures.

**Figure 4.**
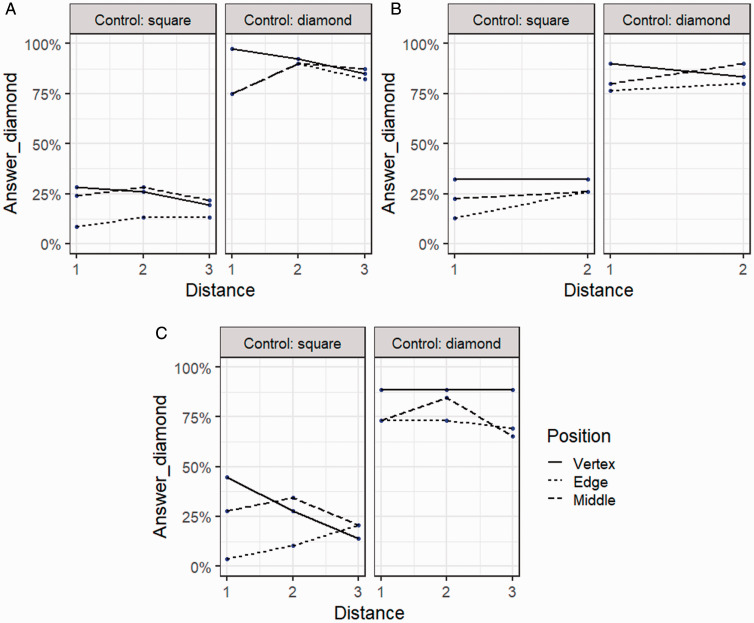
The survey-based response percentages of the figure shape being perceived as a diamond related to the shape perception of the control stimulus in Parts A (A), B (B), and C (C) of Experiment 1.

Interestingly, the perception of the control figure had the most substantial effect on participant’s overall shape perception in all parts of the forced-choice task. Figure 4 demonstrates how the responses of shape perception are changed according to the response given to the control stimulus (rotated square). If the control stimulus was considered as a square, the other stimuli with the additional element were more likely to be perceived as squares as well. While if the control stimulus was recognised as a diamond, the other stimuli with the additional element were more likely to be perceived as diamonds.

## Experiment 2

### Stimuli

In Experiment 2, the same stimuli were presented as in Experiment 1, but the subsequent analysis of the results included only data viewing the stimuli in Parts A and C (see Figure 1A and C). Each of three parts was demonstrated in randomised order. The results of gaze parameters in Part B of Experiment 2 where the additional element was located inside the figure were removed from further data analysis as it was not possible to determine whether the gaze is directed to the additional element or whether the whole figure is viewed as a whole.

### Method

Experiment 2 involved binocular eye tracking and the given task was only to view the stimuli presented on the computer screen (free viewing with no additional instructions). Eye movement data were collected using IViewX RED500 eye tracker (500 Hz, SMI-SensoMotoric Instruments, Germany).

**Figure 5.**
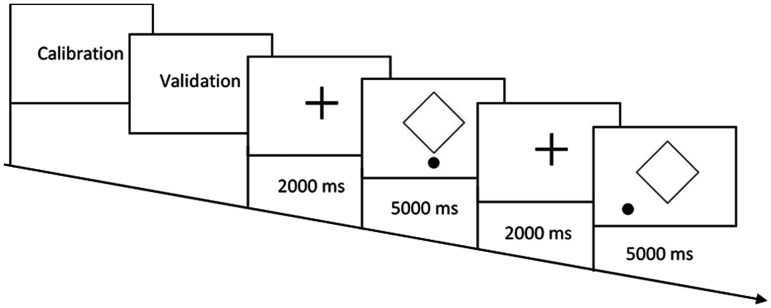
Schematic representation of Experiment 2 sequence.

To obtain accurate eye movement data, a binocular 5-point calibration method in the centre of the screen (30° horizontally and 20° vertically) and validation were performed before Experiment 2. All three parts of Experiment 2 were presented in a randomised order. Each stimulus was presented for 5000 milliseconds. All the stimuli of each part of Experiment 2 were also presented in a randomised order. Before each stimulus, a fixation cross was presented for 2000 milliseconds at the centre of the screen (see Figure 5). Participants were asked to fixate their gaze on the fixation cross when it was displayed, but at the time of the stimulus presentation, participants were free to view and reallocate their gaze to the points of interest on the computer screen.

### Results

**Figure 6.**
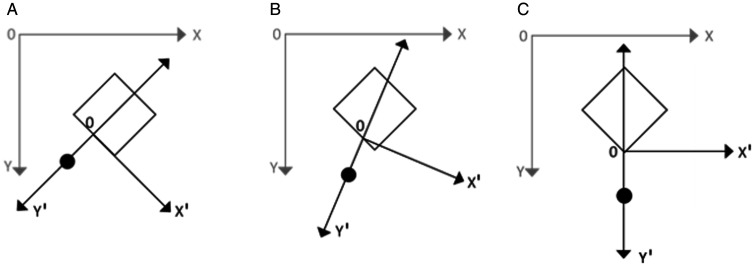
Illustrative example of coordinate transposition for 45° rotation (A), 22.5° rotation (B), and without rotation (C). The grey coordinate system represents originally obtained coordinates with the origin at the top left corner of the screen. The black coordinate system represents new coordinates with the origin on the tetragon edge or vertex that is closest to the dot position.

**Figure 7.**
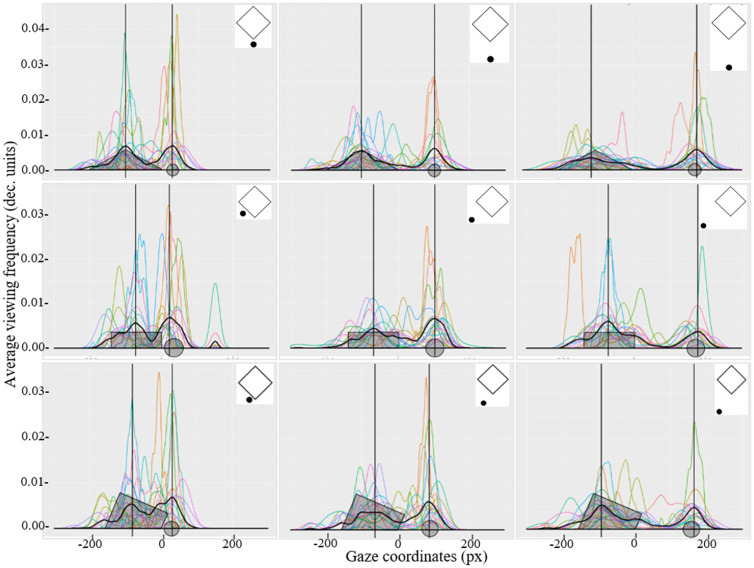
The nonparametric density of transposed *y*-axis coordinates representing the gaze direction in one dimension when attending the stimuli presented in Part A of Experiment 2. Each curve represents the individual results of each participant, and the thicker black curve represents the average gaze distribution for all participants. Vertical lines indicate the position of maximum values for the average gaze distribution for all participants. Location of the figure and the additional element are marked in the coordinate plane (grey areas and the grey circles on *x*-axis).

**Figure 8.**
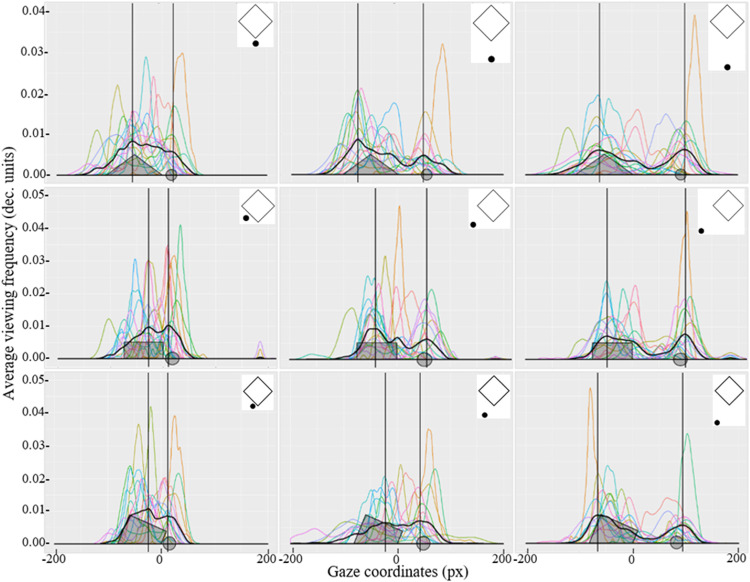
The nonparametric density of transposed *y*-axis coordinates representing the gaze direction in one dimension when attending the stimuli presented in Part C of Experiment 2. Each curve represents the individual results of each participant, and the thicker black curve represents the average gaze distribution for all participants. Vertical lines indicate the position of maximum values for the average gaze distribution for all participants. Location of the figure and the additional element are marked in the coordinate plane (grey areas and the grey circles on *x*-axis).

The aim of Experiment 2 was to determine whether participant’s gaze was directed towards the figure, the additional element or towards somewhere in the area between both objects. To simplify the analysis of the results, the recorded two-dimensional data (of actual gaze position on the screen) were reduced to one dimension by projecting it on the axis that joins the additional element with the centre of the figure. Technically, the new coordinate is acquired by 45 (for stimuli with an additional element located at the edge) and 22.5° rotation (for stimuli with an additional element at the middle position), translation of the origin to the tetragon side or vertex closest to the additional element, and subsequent point projection on the ordinate axis (i.e., the origin of the rotated system is on the closest point on the edge or at the closest vertex; see Figure 6). Finally, each participant’s gaze frequency data on the new *y*’ axis were represented by its nonparametric density, see Figures 7 and 8.

Using RStudio (R Core Team, Vienna, Austria), the data were plotted graphically using the ggplot function, which represents the average gaze distribution density of each participant with the geom_density function and the average gaze distribution of all participants (see Figures 7 and 8). The average frequency of transposed *y*-axis coordinates was analysed to determine which parts of the demonstrated stimuli the gaze was most frequently directed to (i.e., how often the direction of view was turned in the specific transposed *y*-axis coordinates). In Parts A (see Figure 7) and C (see Figure 8) of Experiment 2, only stimuli with the additional element were analysed. Stimuli without the additional elements were considered only as control stimuli and were removed from further data analysis.

To analyse the results, blinks were removed from the resulting data. Eye movement data coordinates obtained during the first 200 milliseconds of each displayed stimulus, which corresponds to saccade latency time, were also removed from further analysis (i.e., gaze parameters were analysed starting from the stimulus demonstration 200 milliseconds).

The average gaze distribution of all participants indicates that with increasing distance between the two elements, the gaze is more directed towards the objects and is less located in the area between the objects. Interestingly, in Part A where the stimuli were larger in size, the tendency to direct gaze towards the COG (i.e., in the space between both stimuli) was not observed. However, when the stimuli are smaller in size (as in Part C of Experiment 2), a larger proportion of fixations that are located in the area between the two stimuli can be observed.

By finding the maximum values for gaze distribution and knowing the position of the figure and the additional element in the coordinate plane, it was possible to observe that in Part A of Experiment 2, the maximum values are located at or very near to the centre of a tetragon and the dot position. When finding the maximum values for the stimuli demonstrated in Part C of the experiment, another relation could be observed from gaze distribution graphics: As the distance between the figure and the additional element increases, gaze not only was more directed to the objects but also was located further away from the centre of each element. Perhaps, this tendency can be ascribed to measurement errors which were *x* = (0.38 ± 0.02°) and *y* = (0.30 ± 0.02°) for the Part C stimuli and *x* = (0.39 ± 0.03°) and *y* = (0.34 ± 0.03°) for the Part A stimuli. These measurement errors correspond to approximately 12 to 14 px in the coordinate plane. This measurement error corresponds to half of the diameter of the additional element in Part C and one third of the diameter if the additional element in Part A. Thus, these small deviations from the centre of the elements can be attributed to a measurement error, and it cannot be claimed that the participant did not look directly at the centre of these elements.

**Figure 9.**
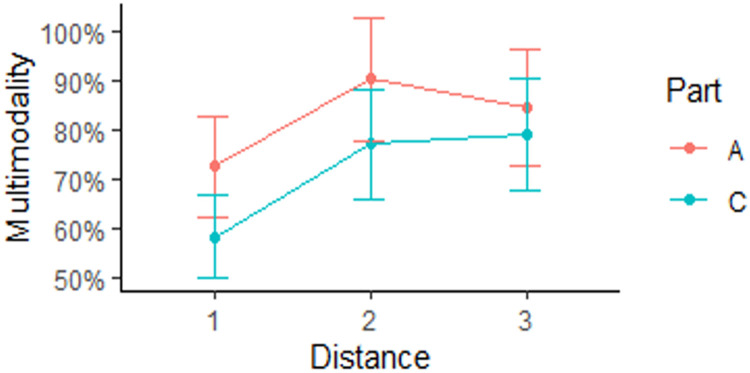
Percentage of cases where the gaze distribution was multimodal depending on additional element’s distance for Parts A and C of Experiment 2.

To determine whether the increasing distance between the object, the additional element, and the overall stimulus size impacts the gaze distribution, a mixed effects logistic regression model was performed for the response variable of gaze modality. Gaze distribution of each participant for a particular stimulus was summarised by a binary variable, where “0” indicates unimodality and “1” indicates multimodality, using Hartigan’s dip test for unimodality (Hartigan & Hartigan, 1985). The percentage of cases where the gaze distribution was deemed as multimodal is demonstrated in Figure 9.

Figure 9 demonstrates that in both parts of Experiment 2 (A and C) at Distance 1, the percentage of cases where the gaze distribution was multimodal was lower when compared with Distances 2 and 3. Figure 9 also demonstrates that in Part C of the experiment, there were less cases of distribution being multimodal when compared with Part A. Overall, for all participants and all stimuli, there were 82.4% of cases where the distribution was multimodal in experiment Part A, while only 71.5% of distributions were multimodal in Part C.

A mixed effects logistic regression model was used to test the factors impacting the shape of the gaze distribution, employing the binary variable of multimodality as the model response. The full model included fixed effects distance, position type and stimulus size (experiment Parts A and C), and random effect for the survey participant. The likelihood ratio test for the difference of the deviance scores between the nested models was used to determine the significance of the model terms. It was revealed that the position effect was not significant, χ^2^(2) = 1.6, *p* = .452. The effects of stimulus size and distance were significant, χ^2^(1) = 6.2, *p* = .013, and χ^2^(2) = 16.9, *p* < .001, respectively. The interaction effect of distance and size was not significant, χ^2^(2) = 1.2, *p* = .548.

Helmert contrast analysis for the distance revealed that there was a significant difference between the multimodality rates at Distance 1 versus Distances 2 and 3 (*p* < .001), however, no significant difference between Distances 2 and 3 (*p* = .63). Finally, the estimated logistic mixed effects model regression coefficients for the first distance contrast were 0.50 and −0.94 for the smaller stimulus size (experiment Part C). Thus, we see that participants gaze distribution was more likely to be multimodal when additional element’s position was 2 or 3 and less likely to be multimodal when stimulus size was smaller (experiment Part C). Moreover, we can see that the effect of stimulus size was larger than that of the distance.

## Discussion

According to the results obtained in this study, two previously described core principles of visual grouping—proximity and directional organisation—impact shape perception in the case of ambiguous stimuli. Based on the principles of visual grouping and gaze processing in visual grouping, it was hypothesised that the placement of an additional element at different locations might have an impact on the shape assignment and furthermore may influence the resulting shape perception (i.e., diamond or square). The results demonstrate the effect of grouping on the shape perception previously described by Pinna (2010, 2015), indicating that an additional element can affect the perception of a tetragon by perceiving it as a diamond or a square depending on the position of the additional element. Furthermore, the grouping effect tends to become weaker with increasing distance between the two objects, indicating that the increasing distance supports the individuation of two different objects instead of showing grouping effects, which is the case when the distance between both objects is decreased.

Interestingly, the participants’ responses were not only influenced by the resulting grouping effect, but also by the way they perceive the figure without the additional element, that is, their previous experience and knowledge. If the rotated square without the additional element was perceived as a square, it was more likely that by also adding the element the object will still be perceived as a square, and vice versa. While there have been studies demonstrating the relationship between previous experience and the task performance (e.g., Stroop effect: Hodgson et al., 2009; MacLeod, 1991; Stroop, 1935; studies on expert advantage: Abernethy et al., 1994), to our knowledge this is among the first studies indicating that shape assignment is constrained by the initial interpretation (which reflect the effects of previous experience and knowledge of the observer).

Additional factors such as visual impairment, occupation, and other observer-based factors might eventually also affect the shape perception and gaze parameters. However, this is outside of the scope of this study. The language of instruction provided for the tasks could also influence the results obtained by comparing them with similar studies because in English participants may perceive the words *square* and *diamond* differently than the corresponding words in Latvian. In Latvian, the default interpretation would be “kvadrāts” (square) or “rombs” (rhombus, diamond). Notwithstanding the language differences, distance between the figure and the dot (and the figure alone) can be more ambiguous or more biased towards one of the interpretations (i.e., square or diamond).

The results of eye movement analysis support the findings obtained in the forced-choice task, indicating that in the cases when both objects are located closer to each other, gaze distribution is more likely to be unimodal. Thus, in these cases, the presented stimuli are perceived as relatively single objects, while with increasing distance, the gaze distribution is more likely to be multimodal and both elements are attended separately.

Although the results of the forced-choice task did not indicate differences between the shape assignment task based on stimulus size, eye movement analysis clearly demonstrates that stimuli that are smaller in size are more likely to be attended as grouped together (i.e., the gaze distribution is more likely to be unimodal and directed to the area between the stimuli). A stronger effect on grouping occurs in the case of a smaller stimulus (i.e., a smaller element seems to be easier to group together with the also smaller main object) which is also in line with the results of the studies of Im et al. (2016) and Grassi et al. (2016).

The differences between the results of the forced-choice task and the eye movement experiment regarding the stimulus size might be related to several factors. First, because the three parts of Experiment 1 were not in a progressive size, the applied method for analysing the results of the forced-choice task did not include the stimulus size as a dependent variable. Thus, no statistical analysis was made to compare these results directly. It was only observed that the effect of the location and the interaction between the location and the distance of the additional element was significant in both parts (A and C) of the experiment where the same stimuli were different in size. Second, it is also possible that in task-dependent conditions gaze direction would be different, for example, if participants would have been asked to analyse what the figure is, gaze direction might be different as well.

A final issue relevant to the current work is the question whether the ambiguity between diamond and square is perceptual or rather cognitive. The answer to this question depends on the framework that we adopt—either we assume that perceptual and cognitive processes are essentially two parts of the same continuum (e.g., Goldstone & Barsalou, 1998, Goldstone et al., 2015) or we assume that these are two qualitatively different sets of processes (e.g., Firestone & Scholl, 2016; from a point of view of a canonical argument in cognitive science: Fodor, 1983). Although we tend to accept the first framework, the question whether the ambiguity is of a perceptual or cognitive nature is a crucial one in both frameworks of defining perception and cognition. According to our results, the first and initially assigned interpretation impacts the later interpretation of the figure, that is, whether the figure will be seen as a diamond or a square seems to depend on the initial interpretation. This means that the ambiguity between diamond and square is rather a cognitive phenomenon instead of a purely perceptual one as the later assigned interpretation seems to be at least to some extent learned during the initial interpretation. Although the question of what determines the initial interpretation is outside of our research scope, we can certainly agree that this ambiguity is determined by learning and previous experience and that top-down effects from the initial interpretation are at least codetermining later. But taking into account the learning effects, we can still observe changes in perceptual grouping once the independent variables (e.g., distance between figure and dot) are changed; therefore, perceptual processes are at least complimentary in processing this ambiguity (For knowledge effects in perceptual ambiguity, cf. also Girgus et al., 1977; Rock et al., 1994).

Furthermore, an interesting issue is the closer characteristics of this ambiguity. Although perceptual ambiguity can be observed in directional orientation of constituting elements (Attneave, 1968) and our results from modulating distance between the figure and the additional element support this, our results also show that the involved interpretations (square or diamond) are gradual and not discrete. Instead of having two mutually excluding interpretations, we demonstrate that by modulating distance we can achieve a more or less strong case of *square* or *diamond* (and, of course, a stronger case of ambiguity).

## Conclusion

Visual grouping and shape assignment are only one set of factors determining the perception of shape. According to our results, if only visual grouping and shape assignment are taken into consideration, we cannot argue that the additional element unambiguously changes the resulting perception of the shape (i.e., whether the figure is perceived as a diamond or a square). The location and proximity are additional factors that affect visual grouping. On the other hand, the resulting shape perception of the figure is also determined by the perceptual decision when the figure is seen alone without the additional element.
